# Characterization of fatal injuries in oil and gas industry-related helicopter accidents in the Gulf of Mexico, 2004–2014

**DOI:** 10.1186/s40621-020-00288-5

**Published:** 2020-12-07

**Authors:** Kristin Yeoman, Mary B. O’Connor, Sara Sochor, Gerald Poplin

**Affiliations:** 1 National Institute for Occupational Safety and Health, Centers for Disease Control and Prevention, 315 E. Montgomery Ave., Spokane, WA 99207 USA; 2 University of Virginia Center for Applied Biomechanics, 404 Lewis & Clark Drive, Charlottesville, VA 22911 USA

**Keywords:** Aircraft, Accident, Safety, Wounds and injuries, Protective devices, Drowning, Helicopter

## Abstract

**Background:**

Transportation events are the most common cause of offshore fatalities in the oil and gas industry, of which helicopter accidents comprise the majority. Little is known about injury distributions in civilian helicopter crashes, and knowledge of injury distributions could focus research and recommendations for enhanced injury prevention and post-crash survival. This study describes the distribution of injuries among fatalities in Gulf of Mexico oil and gas industry-related helicopter accidents, provides a detailed injury classification to identify potential areas of enhanced safety design, and describes relevant safety features for mitigation of common injuries.

**Methods:**

Decedents of accidents during 2004–2014 were identified, and autopsy reports were requested from responsible jurisdictions. Documented injuries were coded using the Abbreviated Injury Scale (AIS), and frequency and proportion of injuries by AIS body region and severity were calculated. Injuries were categorized into detailed body regions to target areas for prevention.

**Results:**

A total of 35 autopsies were coded, with 568 injuries documented. Of these, 23.4% were lower extremity, 22.0% were thorax, 13.6% were upper extremity, and 13.4% were face injuries. Minor injuries were most prevalent in the face, neck, upper and lower extremities, and abdomen. Serious or worse injuries were most prevalent in the thorax (53.6%), spine (50.0%), head (41.7%), and external/other regions (75.0%). The most frequent injuries by detailed body regions were thoracic organ (23.0%), thoracic skeletal (13.3%), abdominal organ (9.6%), and leg injuries (7.4%). Drowning occurred in 13 (37.1%) of victims, and drowning victims had a higher proportion of moderate brain injuries (7.8%) and lower number of documented injuries (3.8) compared with non-drowning victims (2.9 and 9.4%, respectively).

**Conclusions:**

Knowledge of injury distributions focuses and prioritizes the need for additional safety features not routinely used in helicopters. The most frequent injuries occurred in the thorax and lower extremity regions. Future research requires improved and expanded data, including collection of detailed data to allow characterization of both injury mechanism and distribution. Improved safety systems including airbags and helmets should be implemented and evaluated for their impact on injuries and fatalities.

## Background

Helicopter crashes are a source of morbidity and mortality in several industries, including military, oil and gas, and emergency medical operations. Commercial helicopter accident rates in the U.S. have remained flat during 2006—2015, with an average of 6.1 fatal accidents (range 4–8) per year during this period (AOPA Air Safety Institute, 2018). A total of 178 helicopter crashes occurred in oil and gas operations in the Gulf of Mexico during 1983–2009, resulting in 139 fatalities (Baker et al. 2011). The number of crashes increased from an average of 5.6 crashes per year during 1983–1999 to 8.2 crashes per year during 2000–2009 (Baker et al. 2011). Helicopter crashes in the U.S. oil and gas industry contribute to high fatality rates in the industry. During 2003–2010, the fatality rate for workers in the oil and gas industry was seven times that of all industries. Most offshore fatalities were caused by transportation events, and 75% of the transportation events were related to helicopter accidents, resulting in 49 fatalities (Gunter et al. 2013). Similar to increases in helicopter accidents seen in the oil and gas industry, an increase in the number of medical helicopter accidents was seen during 1993–2002, resulting in 72 fatalities and 64 injuries (Bledsoe and Smith 2004). These results indicate that more research is needed to prevent fatalities and injuries from helicopter crashes in these industries, and implementation and evaluation of interventions such as more comprehensive restraints and helmets should be considered.

Most research on helicopter crashes has focused on helicopter design to improve crashworthiness or on investigations of the underlying causes of crashes. Baker et al. (2011) studied helicopter crashes in the oil and gas industry during 1983–2009, concentrating on the underlying cause of the crashes. Mechanical failures were the leading cause, followed by inclement weather (Baker et al. 2011). The U.S. Army has concentrated on the development of crashworthy helicopters to prevent fatalities (Shanahan 1993; Carper et al. 1983; Gatlin et al. 1971). In an Army report, Shanahan (1993) noted that injuries could be prevented by appropriate helicopter design to improve the strength of the cockpit and cabin, use of restraints, energy absorption capacity, and mitigation of local environmental factors (i.e., placement of objects that could cause injury near seats) and post-crash factors (i.e., extrication after water landing). Shanahan and Shanahan (1989) and Knapp et al. (1978) found a lack of thermal injury in survivable crashes attributable to the requirement for crashworthy fuel systems in Army helicopters. Although crash-resistant fuel systems in civil helicopters may not be as effective as those used in military helicopters, Hayden et al. (2005) showed effectiveness in prevention of post-crash fires and thermal fatalities. Findings from additional research on fuel systems, crash-resistance, seats, and restraints used in military helicopters formed the basis for development and evaluation of criteria for civil helicopters (Jackson 2018; Lee et al. 2009; Desjardins 2006; Robertson et al. 2002; Coltman 1994; Coltman et al. 1985).

Research on helicopter safety has also focused on the impact of drowning in helicopter accident fatalities, a critical issue given that offshore activities produced almost 30% of global output in the oil and gas industry in 2015 (EIA 2016). Helicopter accidents in water have resulted in substantial drowning-related fatalities (Bolukbasi et al. 2011; Brooks et al. 2008; CAA 2003). Factors that contribute to drowning after water impacts include injuries that restrict occupant mobility and performance, water conditions and temperature, and entrapment from blocked exits or inability to release restraints (Chen et al. 1993). Other important factors include helicopter sinking and inversions and insufficient warning time (Brooks et al. 2008; Brooks et al. 2014).

Although previous research has focused on prevention of helicopter crashes, little is known about the injuries sustained when these crashes do occur, in contrast to motor vehicle crashes. The incorporation of seat belts, energy-absorbing steering columns, and other safety features have been very effective in improving motor vehicle crashworthiness (O’Neill 2009). Motor vehicle safety and design improvements are acknowledged to have saved thousands of lives, and studies have estimated that incorporation of even more advanced safety systems could prevent most motor vehicle crash (MVC) fatalities as well (Ryb et al. 2011; Robertson 2007; Farmer and Lund 2006).

In contrast to motor vehicle safety, recent oil and gas helicopter safety efforts have focused on satellite-based surveillance technology. Other safety efforts include provision of weather information, mitigation of methane gas ingestion, and improvements in training, equipment, safety management, and operational control (BSEE 2015; FAA 2012; Tippee 2009; Stevens and Sheffield 2006). A multifaceted program using a satellite-based tracking system was instituted in 2009 by the Federal Aviation Administration (FAA), the Helicopter Safety Advisory Conference (HSAC), the oil and gas industry, commercial technology contractors, Helicopter Association International (HAI), and many of the helicopter operators in the Gulf of Mexico (Gray 2018; FAA 2017). This technology allowed controllers to observe air traffic over the Gulf of Mexico, more efficiently separate traffic, and speak with pilots directly. To provide pilots with real-time weather information, weather sensors were also installed at many of the platforms (FAA 2010). Since implementation of this technology and increased industry safety efforts, there has been a decrease in accidents and fatalities in Gulf of Mexico helicopter operations. During 2009–2013, 21 accidents with 11 fatalities occurred, whereas during 2014–2018, nine accidents resulted in four fatalities (HSAC 2019a; 2015; 2011). During 2009–2013, the oil industry helicopter accident rates per 100,000 flight hours in the Gulf of Mexico were 2.32, 0.0, 1.58, 1.58, and 0.98, respectively, whereas the five-year accident rate in 2018 was 0.83 (HSAC 2019a; HSAC 2011). The satellite technology allowed for rapid recognition of incidents and more efficient search and rescue operations. Thus, workers surviving an aircraft crash with limited injuries could be more likely to survive with expedited rescue. However, this technology does not prevent or mitigate injuries from the crashes that do occur. Along with knowledge of impact conditions and injury mechanisms, knowledge of helicopter crash-related injury distributions could facilitate development and implementation of safety improvements to reduce the risk of common injuries and fatalities resulting from helicopter crashes, similar to design modifications that have occurred in motor vehicles as a result of injury research. In contrast to the specific and detailed descriptions of injuries incurred in MVC events, prior helicopter research has classified injuries into very broad environmental and traumatic categories, with traumatic injuries further classified into contact and acceleration injuries (Shanahan 1993). Although this classification system might be useful in helicopter engineering and design focused on crashworthiness and structural integrity, it does not provide relevant information for the specific injury distributions that should be addressed when considering additional safety features not currently or routinely used in helicopters. Knowledge of injury distributions with crashworthiness is essential to identify mechanisms of injury, prioritize the most common or impactful injuries, and justify the implementation of interventions.

Therefore, we aimed to describe the distribution of injuries found among decedents in helicopter accidents in the oil and gas industry and to summarize literature on safety measures that should be considered to mitigate injury severity in helicopter crashes. Although research has been conducted in the area of crash prevention, we focused on injuries rather than the underlying causes of the crashes. We focused on helicopter accidents related to the oil and gas industry because helicopters are used extensively in this industry to transport workers and cargo to offshore, remote, and diverse locations around the world. We analyzed data from accidents in the Gulf of Mexico because oil and gas companies operating in the Gulf of Mexico are represented by organizations promoting robust aviation safety programs (HSAC 2019b; IAOGP 2019; USHST 2019; HAI 2018), allowing us to concentrate on fatalities that occurred despite high safety standards. To focus future research and recommendations for injury prevention and post-crash survival, we performed a descriptive analysis of injury distributions and compared injury frequency among drowning and non-drowning victims. We also provided a more detailed injury classification of moderate or worse (AIS 2+) injuries in order to identify potential areas of enhanced safety design.

## Methods

Helicopter accidents in the Gulf of Mexico during 2004–2014 were identified from the National Transportation Safety Board’s (NTSB)‘s Aviation Accident Database. The NTSB is charged with investigating and determining the probable cause of civil aviation accidents. An accident is defined by 49 CFR Part 830 as “an occurrence associated with the operation of an aircraft which takes place between the time any person boards the aircraft with the intention of flight and all such persons have disembarked, and in which any person suffers death or serious injury, or in which the aircraft receives substantial damage” (Notification and reporting of aircraft accidents or incidents, 49 C.F.R. § 830 2019). The NTSB database contains information about each accident, including an event narrative providing a statement of facts, conditions and circumstances pertinent to the incident; information about the pilot, the aircraft, and known weather conditions; and probable cause of the accident. Accident reports were reviewed manually for determination of inclusion in the study. Criteria for inclusion included the following: the operation was conducted in support of the oil and gas industry, the accident occurred in a helicopter in the Gulf of Mexico, and the accident resulted in at least one fatality from crash-related injuries. To identify accidents meeting these inclusion criteria, the NTSB database was searched for fatal helicopter accidents with location listed as the Gulf of Mexico or any surrounding state (Florida, Alabama, Mississippi, Louisiana, and Texas). All NTSB factual report narratives were reviewed for information confirming that helicopter departure or destination sites were oil and gas industry offshore platforms, helipads, or vessels; and that cargo or passengers were owned or employed by oil and gas companies. In addition to NTSB reports, information was obtained from newspaper articles and the NTSB’s public docket created for each accident, which may contain witness statements, pictures, and other information relevant to the NTSB’s investigation.

Names of pilots and passengers from each accident and medical examiner contact information were obtained from media reports or the FAA in order to request autopsies. In the United Sates, autopsies are performed on pilots killed in fatal aviation accidents by the medical authority in the jurisdiction where the accident occurred. In addition, autopsies are often performed on other persons killed while working or traveling to or from their place of work. Autopsy reports were requested from medical examiners/coroners based on the jurisdictions reported by the NTSB as responsible for performing autopsies.

Autopsy reports were reviewed by an AIS Certification Board Certified Abbreviated Injury Scale Specialist (CAISS), and each documented injury was hand coded using the Abbreviated Injury Scale (AIS) 2005 Update 2008 (AAAM 2008; Gennarelli and Wodzin 2008). The AIS is an anatomically based, consensus-derived, global severity scoring system that provides a link between injury descriptions and injury severity (AAAM 2008; Gennarelli and Wodzin 2008). AIS codes are composed of a unique numerical identifier that includes information on affected body region/anatomical location and injury severity. Each injury was coded into AIS body region categories based on injury location as follows: head, face, neck, thorax, abdomen, spine, upper extremity, lower extremity, and external/other. The external/other category includes burns and drownings. Injury severity was coded on a six point ordinal AIS scale as follows: 1 = minor, 2 = moderate, 3 = serious, 4 = severe, 5 = critical, 6 = maximal, and 9 = unknown severity (AAAM 2008; Gennarelli and Wodzin 2008).

Descriptive statistics were calculated based on AIS body region and severity categories. Frequency and proportion of injuries by AIS body region and severity were calculated among all injuries and after categorization into minor (AIS 1), moderate (AIS 2), and serious or worse (AIS 3+) injuries. Individual injuries were then grouped into more detailed body region categories, because examining injury distributions according to AIS body region and severity may not be specific enough for identifying targeted design and injury control strategies from an engineering or biomechanics standpoint. However, a direct listing of specific AIS codes would be overly detailed. Similar to a recent investigation of injury trends in belted MVC occupants, injuries of moderate or worse severity (AIS 2+) were classified into one detailed body region category based on research from anatomy, injury biomechanics, and injury epidemiology in order to help identify focused areas of prevention and control (Forman et al. 2019). Moderate or worse (AIS 2+) injuries were chosen for this classification system because critical injuries that cause morbidity and mortality and are preventable through engineering designs are included in these severity categories. Frequency and proportion of injuries by these more detailed body regions were calculated. From autopsy reports, decedents were categorized as having drowned or not. Average number of injuries were compared between drowning and non-drowning victims as well as pilots and passengers. All analyses were performed in Stata version 15.1.

## Results

A total of 14 fatal helicopter accidents occurring in the Gulf of Mexico during 2004–2014 and meeting study criteria were identified in the NTSB database, resulting in 42 fatalities. Among the decedents, 14 were pilots and 28 were passengers. Age range was 23–66 years, with a mean of 40.9 years. Autopsy reports from the 35 male decedents involved in 11 helicopter crashes were obtained. Autopsies were not conducted on four decedents from two accidents, and the bodies of three decedents from one accident were not recovered. In addition to the 42 fatalities, five of the 14 accidents with fatalities also had at least one survivor (*N* = 12). Detailed information on survivors’ injuries was not available for analysis.

**Fig. 1:**
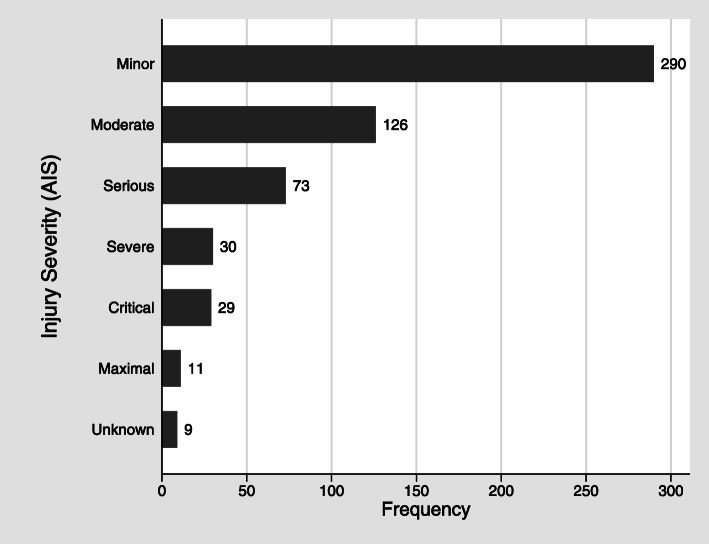
Frequency of injuries by Abbreviated Injury Scale (AIS) injury severity, *n* = 568

Among 35 decedents on whom autopsies were conducted, 568 injuries were documented in autopsy reports. Decedents had a median of 12 injuries documented, with a range of 1–44 injuries. The majority (51%) of injuries were minor, and the frequency of injuries decreased as the injury severity score increased (Fig. 1). Nine (1.6%) injuries were coded as unknown severity because they lacked adequate information. As expected, the highest individual AIS severity score was 5 or 6 in a majority (71.4%) of decedents (Table 1). However, the highest AIS scores were below the critical or maximal severity levels in 28.6% of decedents (Table 1). Of these, 20% were drowning victims.

**Table 1:** Frequency and proportion of decedents’ highest Abbreviated Injury Scale (AIS) injury severity score

AIS severity	AIS severity score	Frequency	Proportion
Critical or maximal	AIS 5–6	25	71.4%
Severe	AIS 4	5	14.3%
Serious	AIS 3	3	8.6%
Moderate or minor	AIS 1–2	2	5.7%

The percentage of minor (AIS 1), moderate (AIS 2), and severe or worse (AIS 3+) injuries differed by body region, with 93.4% of injuries of the face and neck being minor, and 53.6% of injuries to the thorax being serious or worse (Table 2). Among abdominal injuries, 46.2% were minor, 32.7% were moderate, and 21.2% were serious or worse, whereas among spine injuries, 0 were minor, half were moderate, and half were serious or worse (Table 2). Minor injuries were most prevalent in the face, neck, upper and lower extremity, and abdominal regions, whereas serious or worse injuries (AIS 3+) were most prevalent in the thorax, spine, head, and external/other regions (Table 2). Fewer external/other and spine injuries were reported on autopsies, but the injuries tended to be more serious. Thorax injuries were high in both frequency and proportion of serious (AIS 3+) injuries.

**Table 2:** Abbreviated Injury Scale (AIS) body region frequency and proportion for injuries of various severities

AIS body region	All injuries^**a**^	Minor (AIS 1)	%	Moderate (AIS 2)	%	Serious or worse (AIS 3+)	%
**Head**	51	15	31.3	13	27.1	20	41.7
**Face**	76	71	93.4	5	6.6	0	0.0
**Neck**	11	10	90.9	0	0.0	1	9.1
**Thorax**	125	27	21.6	31	24.8	67	53.6
**Abdomen**	52	24	46.2	17	32.7	11	21.2
**Spine**	23	0	0.0	9	50.0	9	50.0
**Upper extremity**	77	57	74.0	19	24.7	1	1.3
**Lower extremity**	133	81	61.4	32	24.2	19	14.4
**External/other**	20	5	25.0	0	0.0	15	75.0

^a^Numbers don’t always add up from minor, moderate, serious/worse because of unknown injury codes

**Fig. 2:**
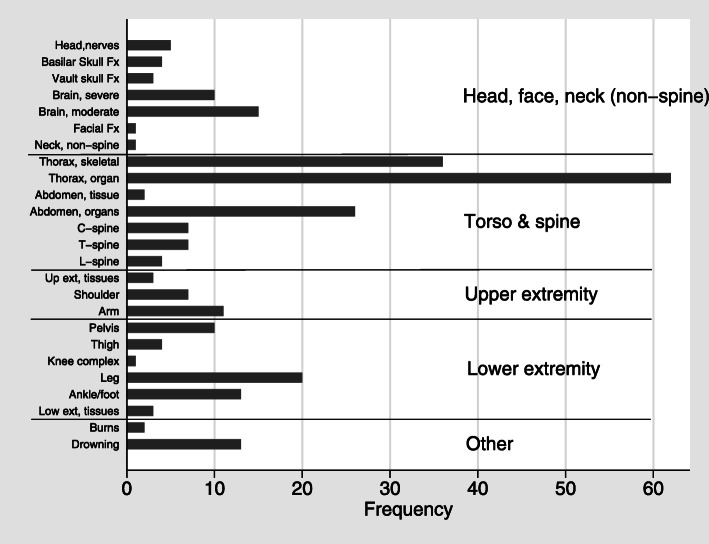
Frequency of moderate or greater (Abbreviated Injury Scale 2+) injuries, using more detailed body regions

As illustrated in Fig. 2 describing injuries by more detailed body regions, moderate and severe brain injuries were the most common injuries seen in the head, face, and neck. Injuries to the thoracic and abdominal organs predominated among injuries to the torso and spine, followed by injuries to the thoracic skeletal structures. Among upper extremity injuries, arm and forearm injuries were most common, whereas leg (i.e. tibia and fibula) injuries were most common among lower extremity injuries.

Drowning was noted in 13 (37.1%) of 35 fatalities. Among these 13 drowning victims, 173 injuries of all severities (AIS 1–6) were documented for an average of 13.3 injuries per victim, whereas the 22 non-drowning victims averaged 18.0 injuries per individual (*n* = 395 injuries). After removing the drowning-specific and minor injury codes, drowning victims averaged 3.8 documented injuries per victim, whereas non-drowning victims averaged 9.4 injuries per victim; no significant difference in mean AIS was seen between persons who drowned (AIS 2.98) and those who did not (AIS 2.88). Comparing detailed injury classifications between drowning and non-drowning fatalities, drowning victims had a higher proportion of moderate brain injuries (17.6% vs 2.9%) (Table 3). However, four of the cases were reported as brain edema, which could be caused by (rather than a precipitant of) drowning. After excluding these cases, the proportion of moderate brain injuries among drowning victims decreased to 7.8%. The remaining moderate brain injuries among drowning victims were hematomas or subarachnoid hemorrhages.

**Table 3:** Frequency and proportion of Abbreviated Injury Scale 2+ injuries by detailed body regions^b^ and drowning

Detailed body group	Non-drowning	% (column)	Drowning	% (column)	All	% (column)
**Head, nerves**	4	1.9	1	2.0	5	1.9
**Basilar skull fx**	4	1.9	0	0.0	4	1.5
**Vault skull fx**	3	1.5	0	0.0	3	1.1
**Brain, severe**	7	3.4	3	5.9	10	3.7
**Brain, moderate**	6	2.9	9	17.6	15	5.6
**Facial fx**	1	0.5	0	0.0	1	0.4
**Neck, non-spine**	1	0.5	0	0.0	1	0.4
**Thorax, skeletal**	25	12.1	11	21.6	36	13.3
**Thorax, organ**	51	24.8	11	21.6	62	23.0
**Abdomen, tissue**	2	1.0	0	0.0	2	0.7
**Abdomen, organs**	24	11.7	2	3.9	26	9.6
**C-spine**	4	1.9	3	5.9	7	2.6
**T-spine**	5	2.4	2	3.9	7	2.6
**L-spine**	1	0.5	3	5.9	4	1.5
**Upper ext, tissues**	2	1.0	1	2.0	3	1.1
**Shoulder**	7	3.4	0	0.0	7	2.6
**Arm**	11	5.3	0	0.0	11	4.1
**Pelvis**	8	3.9	2	3.9	10	3.7
**Thigh**	3	1.5	1	2.0	4	1.5
**Knee complex**	0	0.0	1	2.0	1	0.4
**Leg**	19	9.2	1	2.0	20	7.4
**Ankle/foot**	13	6.3	0	0.0	13	4.8
**Lower ext, tissues**	3	1.5	0	0.0	3	1.1
**Burns**	2	1.0	0	0.0	2	0.7
**Total**	206	100.1^a^	51	100.0	257	100.0

^a^Percentages do not add to 100.0 because of rounding

^b^Detailed body region categories based on Forman et al. 2019

Injuries were also compared between pilots and passengers, and the proportional distribution of injuries by body region did not differ between them (graphs not shown). On average, pilots sustained a greater number of injuries (21.3) than passengers (13.9); however, there was no statistically significant difference in the number of injuries, or the average maximum severe injury sustained (5.2 to 4.5, respectively; *p* = 0.10).

## Discussion

Our analysis characterized the injury distribution for fatal helicopter crash victims and demonstrated variations in injury frequency and proportion of minor, moderate, and serious or worse injuries within each AIS body region. The face and upper extremities had a high number of total injuries, but the majority of those injuries were minor. The thorax and lower extremity body regions had the highest frequency of injuries of at least moderate severity. First described by Forman et al. (2019), our analysis also provided a more detailed injury classification of moderate or worse (AIS 2+) injuries in order to help characterize crash biomechanics and identify potential areas of enhanced safety design. For example, as opposed to the AIS body region category “lower extremity”, by distinguishing between leg and pelvic injury severities, one may begin to narrow the impact points and pathways of force distribution, which in theory should help identify the likely biomechanics of injury and target more focused intervention strategies. Because the greater number of body regions resulted in fewer injuries per region, the detailed injury classification system could not be organized into minor, moderate, and serious or worse categories. Rather, all moderate or worse injuries were categorized into one of detailed body region categories. Moderate injuries (e.g. organ lacerations, fractures) were included with serious or worse injuries in this classification system because they contribute to injury morbidity, ability to exit a sinking helicopter, and mortality from multiple coexisting injuries. Furthermore, describing injuries in this manner can more readily identify potential engineering and safety design solutions that can improve their prevention. Among these detailed injuries, organ injuries within the thorax accounted for the greatest number of moderate or worse injuries, followed by thoracic bone, and abdominal organ injuries. Among the lower extremity injuries, lower leg injuries were most common, followed by ankle/foot, pelvis, and thigh injuries. Moderate and severe brain injuries also accounted for a relatively high proportion of total moderate or worse injuries.

Similar to our study, Taneja and Wiegmann (2003) reported that thorax/abdominal organ and lower extremity injuries were common among pilots killed in helicopter crashes, but brain, rib, and skull injuries were also very common. Research in U.S. Army and Navy helicopter crashes during 1985–2005 found that decedents had the highest frequency of injuries to the head and chest, often followed by the spine and lower extremities (Kent 2010; Mapes et al. 2008). An analysis of water-related crashes demonstrated that 19% of injuries were from accelerative forces, and the majority of these were spinal injuries (Chen et al. 1993). Another study of U.S. Army helicopter accidents found differing injury distributions between decedents and survivors, with head, upper torso, lower torso, and lower extremity injuries occurring most frequently among decedents, and lower extremity, head, face, and upper extremity injuries occurring most frequently among survivors (Barth n.d.). Because data on survivors’ injuries were not available, we were unable to compare injury distributions between decedents and survivors. Nevertheless, we have demonstrated high frequency and severity of thorax, lower extremity, and head injuries among helicopter crash fatalities, consistent with other studies.

The injury distributions observed in our study contrast with the distributions often seen in airplane and motor vehicle crashes. An analysis of fatal injuries among pilots in general aviation airplane accidents found that the most common injuries sustained were fractures of the ribs, skull, facial bones, tibia, and pelvis (Wiegmann and Taneja 2003). A study of aviation-related fatalities in 1980 and 1990 demonstrated that 42% of fatalities had multiple injuries, 12% had internal injuries, and 22% had head injuries, but the study did not provide further details on types of internal injuries (Li and Baker 1997). Upper and lower extremities were the most commonly injured body regions among MVC victims with at least one AIS 2+ injury (Forman et al. 2019; Ye et al. 2015; Poplin et al. 2015). However, Mallory et al. (2017) demonstrated injury distributions similar to our analysis, with higher injury rates in the thorax than other AIS body regions for most types of MVC impacts (i.e. rear, rollover, side, frontal oblique, or frontal impacts). Additionally, a majority of fatalities were attributed to head or thorax injuries (Mallory et al. 2017). Injury distributions between similar studies can be difficult to compare because of various analysis methods, with some studies reporting the proportion of persons with specific injuries, other studies reporting the proportion of specific injuries among all injuries, and other studies reporting all injuries or injuries specifically of survivors or decedents. Different versions of AIS coding also affect comparisons between studies. Despite differences in analysis methods, thoracic organ injuries appear to be a more prominent finding in our study compared with those found in fixed wing aircraft and motor vehicle crashes. This difference in thoracic injuries between helicopter and motor vehicle crashes could be related to the use of airbags and belt load limiters in motor vehicles that increase surface area to dissipate crash forces as well as motor vehicle features such as hood crush and steering column stroke/shear capsule to absorb energy. Additionally, crash dynamics and loading in helicopter crashes may be more complex and severe than those in fixed-wing aircraft and motor vehicles.

Knowledge of injury patterns allows investigators to evaluate strategies to reduce morbidity and improve survivability from helicopter crashes. Types of injuries sustained in helicopter crashes depend on a number of factors, most notably the crash mode, speed, position, and orientation of the occupant, along with the safety features of the helicopter. However, occupants of helicopters still die in crashes that are considered survivable based on acceleration forces that are within limits of human tolerance (Dodd 1994; Shanahan 2004). To survive a crash, occupants must survive impact forces, remain capable of egress, and continue survival until rescue. In our analysis, not all decedents had injuries classified as critical or maximal severity, but they still died, most likely as a result of concomitant injuries or drowning. With better protective equipment, some of these decedents might have survived. Restraint technology, use of personal protective equipment, training in helicopter extrication, and standardized record keeping and documentation of circumstantial information and injury details are all areas that should be improved to decrease morbidity and mortality from helicopter accidents. The more detailed body region classification system presented in this analysis aims to identify opportunities for interventions to improve crash survivability. Specifically, restraint systems to decrease torso and lower extremity injuries, helmets to decrease head injuries, and safety equipment to prevent drownings should be evaluated for their impact on injuries and fatalities.

Appropriate restraints for the upper torso immobilize occupants and are essential for survival in a helicopter crash (Bolukbasi et al. 2011; Shanahan 2004). A study of civilian (i.e. non-military) helicopter crashes by Coltman et al. (1985) found that only vertical impact forces exceeded thresholds that would be predicted to cause severe injuries for well-restrained occupants. These forces may be mitigated through energy attenuation to reduce impact loads through crashworthy or stroking seats, which incorporate controlled deformation in a vertical direction, cables and links in restraint systems, crushable subfloor structures, or a combination thereof (Bolukbasi et al. 2011; Coltman et al. 1985). In a study of water-related helicopter accidents, occupants wearing shoulder harnesses and lap belts had fewer serious (AIS 3–6) impact injuries than those wearing lap belts alone (Chen et al. 1993).

Injuries can also occur from direct impact with the helicopter structure or objects within the helicopter, and these contact injuries may be more important than those caused by deceleration forces in survivable crashes (Taneja and Wiegmann 2003). Taneja and Wiegmann (2003) posit that use of shoulder restraints and head protection may significantly influence the pattern of injuries in potentially survivable helicopter accidents. However, availability of these safety features is limited. Labun (2014) found that lap belts, shoulder harnesses, restraints using inertia reels, and seats frequently used by pilots were largely effective, although the same protective equipment is less available and less effective when used by cabin occupants. In our study, no difference in number of injuries or maximum severe injury was seen between pilots and passengers, although insufficient information was available on use of protective equipment between the two groups. Modern restraint system technologies include 4- and 5-point restraint systems with locking inertia reels, shoulder harnesses, airbag restraints, and lap belts.

Airbag restraints are of particular interest given the high frequency of thoracic organ injuries seen in our analysis. Airbags are not effective in situations of collapse or penetration of occupied space, and our data did not include information that could be used to determine the mechanism of thoracic injuries. Studies using crash test dummies have found that airbag restraints provide greater protection than standard restraints (Wright and Albery 2013). Airbags decrease pilot forward motion (Vadlamudi et al. 2011) and can prevent impact with objects or the helicopter structure, reduce slack in restraints, and reduce head movement (Bolukbasi et al. 2011; DOD 2009; Ford 1995). Cockpit airbag systems and belt retractors that position occupants appropriately prior to impact can reduce flailing (Bolukbasi et al. 2011). Decreased severity of head strikes was demonstrated when airbags supplemented restraint systems in U.S. Army helicopters (Alem et al. 1991). Despite these findings, airbags are not readily available in civilian (i.e. non-military) helicopters, and effective restraint systems for all occupants should become a standard in helicopter design (Bolukbasi et al. 2011; Mapes et al. 2008). Airbag restraints did not appear to be available on any aircraft in this study, based on information detailed on the accident/incident report forms, which were updated in 2013 to reflect the potential installation and deployment of inflatable restraints. Collaboration between manufacturers and industry should be fostered to accelerate the development and installation of airbags as standard equipment for helicopters. Further study of the impact of airbags on preventing thorax injuries in helicopter crashes should be considered.

Helmets can supplement restraints and improve injury and fatality rates in helicopter accidents. Crowley (1991) demonstrated that helmets significantly protect occupants from serious head injuries in survivable U.S. Army helicopter accidents. Head injury is the leading cause of injuries and fatalities on U.S. military helicopters (Bolukbasi et al. 2011) and is associated with the greatest risk of death (Kent 2010). Given the frequency of head injuries, head protection is recommended for all occupants of helicopters operated by the U.S. military (Bolukbasi et al. 2011; DOD 2009; Mapes et al. 2008). The same level of protection should be considered for civilian operations as well. In our analysis, head injuries occurred frequently, and further research is needed to confirm our finding of a higher proportion of moderate brain injuries among drowning victims to evaluate whether this is a contributing factor in drownings. Water-based crash victims with brain injuries might be less able to extricate themselves from the helicopter and thus, more likely to drown, especially with concomitant injuries.

If occupants survive the initial impact, the ability to evacuate the aircraft quickly will contribute to further survival of water-based crashes. Previous research demonstrated that accidents occurring in water resulted in a high percentage of drowning-related fatalities (Bolukbasi et al. 2011; Brooks et al. 2008; CAA 2003). We analyzed injuries by drowning status to evaluate disparities in injuries between drowning and non-drowning victims. Although no significant difference was seen between injury severity, drowning victims had fewer documented injuries compared with non-drowning victims. However, medical examiners/coroners conceivably could have provided less detailed examinations for drowning victims after the initial cause of death determination. The higher proportion of moderate brain injuries among drowning victims could be explained in part by drowning-associated brain edema, but moderate brain injuries were still more common among drowning victims after excluding brain edema. Regardless of other injuries, drowning occurred in approximately 37% of decedents in our analysis, and strategies to prevent drowning should be considered. Impact with water presents different challenges to survival than impact with terrain and necessitates a need for rapid egress. Helicopter inversion and sinking contribute to the increased frequency of drowning in survivable incidents (Bolukbasi et al. 2011; Taber and McCabe 2007). Therefore, research should be prioritized on improving egress from sinking helicopters and on the impact of helmet use on preventing drownings.

In addition to difficulties from egress related to injuries, high fatality rates in water-related incidents are also related to difficulties with egress from the sudden rush of water, disorientation, difficulty in visualizing the environment, entanglement with debris, and challenges in releasing restraints and opening doors (Bolukbasi et al. 2011; Brooks et al. 2008). Helicopter underwater escape training (HUET) reduces disorientation and allows occupants to practice aircraft escape from an inverted position in the dark (Bolukbasi et al. 2011). Training is recommended for pilots and personnel who regularly fly offshore for work duties (HSAC 2004). Some studies have reported higher survival of water-based helicopter crashes among persons receiving escape training compared with untrained persons (Cunningham 1978; Hytten 1989). However, research findings are not in agreement regarding the effectiveness of HUET, due in part to lack of training standards, the degree to which simulations reflect the real-world physical and cognitive elements of egress, skill retention, and training recency (Taber 2014; Taber and McGarr 2013). Providing information specific to previous HUET experience in accident reports would provide safety researchers with data to direct efforts. In addition to HUET training, supplemental breathing devices can provide several minutes of air while occupants exit a submerged helicopter. These devices, also called helicopter emergency egress devices, have been used successfully by the U.S. military to increase survivability for occupants in accidents where helicopters are submerged (DOD 2009; Mapes et al. 2008). Given the high number of drownings in our analysis, further research is needed on these and other strategies to prevent drowning.

In addition to consideration of more advanced restraint technologies, use of helmets, and strategies to prevent drowning, standardized record keeping and data collection could facilitate future evaluations of the impact of these safety features on injury severity and mortality from helicopter accidents. NTSB does not investigate injuries sustained in crashes or impact conditions. This information is vital for establishing mechanisms of injury and prioritizing interventions to mitigate these injuries and improve survivability. Data should be collected systematically on both survivors and decedents of helicopter crashes to evaluate patterns of injuries. Autopsies are typically performed when accidents result in pilot or worker fatalities, but documentation of nonfatal injuries sustained and the impact conditions leading to these injuries could be helpful in identifying patterns and intervention strategies for these injuries. During survivor interviews as part of each accident investigation, nonfatal injury results identified by occupant seating location could be obtained and included in the NTSB database. As it is currently constructed, the NTSB dataset does not provide sufficient data for detailed investigations of helicopter crashes, thus limiting the ability to investigate helicopter crashes in depth. In contrast, two research programs within the National Highway Traffic Safety Administration (NHTSA) – the National Automotive Sampling System (NASS) and the Crash Injury Research and Engineering Network (CIREN) – provide detailed data on types and distributions of MVCs, injuries, and their likely mechanisms. Regular and comprehensive review of these data by government, academic, insurance and industry groups has unquestionably led to significant advances in automotive safety, yet comparable data do not exist for helicopter crashes. Without detailed investigations of both fatal and nonfatal injuries in the NTSB database, advances in helicopter safety cannot be made.

NTSB accident reports also need to improve the quality and type of data collected. Accident reports typically include recommendations to prevent future accidents, such as recommendations for requirements for avionics, enhancement of infrastructure, or increased oversight of operations. NTSB investigators have access to information from company records, including training records, interviews with witnesses and survivors, and evidence from investigations and inspections. Including this information on NTSB form 6120.1 (Pilot/Operator Aircraft Accident/Incident Report) could be beneficial in assessing the role of additional training and equipment (NTSB 2013). Specifically, history of water survival training and use of equipment such as restraints, personal flotation devices, life rafts, supplemental breathing devices, and helmets, should be recorded. Additionally, standardized reporting of injuries is needed for autopsies, because no federal regulations exist for autopsy reporting, although practice guidelines for autopsies have been developed by national organizations (NAME 2005; Hutchins et al. 1999).

The oil and gas industry can promote the implementation of additional safety features, support research investigating the impact of these safety features on injuries and fatalities, and promote improved data collection among helicopter accidents involving oil and gas personnel. The influence of the industry and the support of safety and professional trade organizations may be important in improving the quality of data collected by the NTSB in aircraft crashes. Additionally, the industry can investigate the impact of including these safety features on payload, range, cost of operations, and occupant comfort. Operations supporting the oil and gas industry in the Gulf of Mexico were selected for this study based on this highly regulated industry’s focus on safety, comprehensive surveillance, and opportunity for development of meaningful interventions. Industry best practices and guidelines for reducing risk in aviation operations have already been developed and call for higher safety standards than those required by FAA regulations. With the adoption of satellite tracking, location and investigation of accidents occurs quickly and thoroughly. Results of this study can be used by the oil and gas industry to focus attention on aspects of accident investigations and reporting that can improve research and practice in this area. Improvements in aviation operations in the Gulf of Mexico can also be implemented by international offshore helicopter operations and helicopter operations in other industries, and thus the opportunity exists for wide dissemination of research findings and recommendations.

Further research is needed to confirm the findings and generalizability of our study, which was limited by several factors. Information was not available for all decedents on demographics, seat location of deceased passengers within the helicopter, and use of specific safety features such as seat belts or personal flotation devices. Furthermore, flight data recorders were not available or required, and thus information regarding velocity vectors, deceleration rates, and other impact conditions was unknown. Data on impact conditions are crucial to determining mechanism of injury. Although injury distributions are helpful in prioritizing interventions based on the most common and impactful injuries, the scope of the analysis was limited by lack of data on mechanism of injury. Knowledge of both injury distributions and mechanism of injury is essential to recommend the most appropriate interventions. Data on injury distributions were also limited by type (i.e., internal exam vs. external view), quality, and amount of detail of autopsy reports, thus limiting AIS coding of each injury. Nevertheless, the majority of examinations were internal (i.e., dissection of the body); only four (11.4%) examinations were external (i.e., inspection of the outside of the body). Information on survivors was not available, and thus evaluating differences in injury distributions between decedents and survivors was not possible. Sample size was small for detailed body region classifications, limiting comparisons. Finally, our results cannot be generalized to injuries in land-based helicopter crashes.

## Conclusions

This analysis is the first to provide information on injury distributions among helicopter crash fatalities in the Gulf of Mexico supporting the oil and gas industry. Injuries resulting from such crashes often have multiple contributory components, and therefore, multiple opportunities for control strategies. More research is needed to better understand the highest priority injuries on which to focus future design and control strategies. This includes improved data collection that allows for detailed investigations of both injury distributions and mechanisms of injury. This information is required to prioritize and implement the safety features to mitigate the most impactful injuries, both fatal and non-fatal, from helicopter crashes. Our results suggest that further evaluations of airbag restraint systems, helmets, egress training, and supplemental breathing devices to prevent drownings and thorax, lower extremity, and head injuries are warranted. Development, implementation, and evaluation of these and other safety features could effectively decrease morbidity and mortality from helicopter crashes.

## Data Availability

The data analyzed during the current study are not publicly available because autopsy reports were obtained from medical examiners to generate the data and are unavailable for outside use. The NTSB data we used to identify accidents is publicly available.

## Abbreviations

AAAM: Association for the Advancement of Automotive Medicine
AIS: Abbreviated injury scale
AOPA: Aircraft Owners and Pilots Association
CAISS: AIS Certification board certified abbreviated injury scale specialist
BSEE: Bureau of Safety and Environmental Enforcement
FAA: Federal Aviation Administration
HAI: Helicopter Association International
HSAC: Helicopter Safety Advisory Conference
HUET: Helicopter Underwater Escape Training
IAOGP: International Association of Oil and Gas Producers
MVC: Motor vehicle crash
NAME: National Association of Medical Examiners
NHTSA: National Highway Traffic Safety Administration
NTSB: National Transportation Safety Board
USHST: United States Helicopter Safety Team
